# ECG findings in competitive rowers: normative data and the prevalence of abnormalities using contemporary screening recommendations.

**DOI:** 10.1186/2052-1847-7-S1-O19

**Published:** 2015-08-11

**Authors:** MM Wasfy, J DeLuca, F Wang, B Berkstresser, KE Ackerman, A Eisman, GD Lewis, AM Hutter, RB Weiner, AL Baggish

**Affiliations:** 1 Cardiovascular Performance Program, Massachusetts General Hospital, Boston, MA, USA; 2 Harvard University Health Services, Cambridge, MA, USA; 3 Division of Sports Medicine, Boston Children’s Hospital, Boston, Massachusetts, USA; 4 Neuroendocrine Unit, Massachusetts General Hospital, Boston, MA, USA; 5 Cardiovascular Performance Program, Massachusetts General Hospital, Boston, MA, USA

## Background/aim

The international governing body for competitive rowing recently mandated the inclusion of 12-lead ECG during preparticipation screening. We therefore sought to describe normative ECG characteristics and to examine the prevalence of abnormal ECG findings as defined by contemporary athlete ECG interpretation criteria among competitive rowers.

## Methods

Competitive rowers (n=330, 56% male) underwent standard 12-lead ECG at the time of collegiate preparticipation screening. ECGs were analysed quantitatively to develop a sport-specific normative database and then for the presence of abnormalities in accordance with the 2010 European Society of Cardiology (ESC) recommendations and 2013 ‘Seattle Criteria.’

## Results

94% of rowers had one or more training-related ECG patterns including sinus bradycardia (51%), sinus arrhythmia (55%), and incomplete right bundle branch block (42%). Males were more likely than females to have isolated voltage criteria for left ventricular hypertrophy (LVH) (51% vs 8%, p<0.001) and early repolarisation pattern (76% vs 23%, p<0.001). Application of the 2010 ESC criteria, compared to the Seattle criteria, resulted in the classification of a significantly greater number of abnormal ECGs (47% vs 4%; p<0.001). The detection of true pathology, accomplished by both interpretation criteria, was confined to a single case of ventricular pre-excitation.

## Conclusions

Training-related ECG patterns with several gender-based differences are common among competitive rowers. The diagnostic accuracy and down-stream clinical implications of ECG-inclusive preparticipation screening among rowers will be dictated by the choice and future refinement of ECG interpretation criteria.

